# Clinical presentation of eating disorders in young males at a tertiary setting

**DOI:** 10.1186/s40337-015-0075-x

**Published:** 2015-11-09

**Authors:** Chloe Y. Shu, Karina Limburg, Chris Harris, Julie McCormack, Kimberley J. Hoiles, Matthew J. Hamilton, Hunna J. Watson

**Affiliations:** Eating Disorders Program located at Princess Margaret Hospital for Children, Specialised Child and Adolescent Mental Health Service, Child and Adolescent Health Service, Department of Health in Western Australia, Perth, Australia; School of Psychology and Speech Pathology, Curtin University, Perth, Australia; YouthFocus, Perth, Australia; Department of Psychiatry, The University of North Carolina at Chapel Hill, Chapel Hill, USA; School of Paediatrics and Child Health, The University of Western Australia, Perth, Australia

**Keywords:** Child, Clinical presentation, Eating disorders, HOPE Project, Males, Paediatric

## Abstract

**Background:**

Young males with eating disorders are a neglected study population in eating disorders. The aim of this study was to provide knowledge about the clinical presentation of eating disorders in young males.

**Methods:**

The data source was the Helping to Outline Paediatric Eating Disorders (HOPE) Project (*N* ~ 1000), a prospective, ongoing registry comprising consecutive paediatric (<18 years) tertiary eating disorder referrals. Young males with DSM-5 eating disorders (*n* = 53) were compared with young females with eating disorders (*n* = 704).

**Results:**

There was no significant difference in the prevalence of diagnosis of bulimia nervosa (2 % vs 11 %, *p* = 0.26) among sexes. Males had comparable duration of illness (9 months; *p* = 0.28) and a significantly earlier age of onset (*M* = 12 years; *p* <0.001). Shape concern (2.39 vs 3.57, *p* <0.001) and weight concern (1.97 vs 3.09, *p* <0.001) were lower in males, and body mass index *z* score (−1.61 vs −1.42, *p* = 0.29) and medical compromise (odds ratio [OR] = 0.64, 95 % CI: 0.36, 1.12) were comparable. Males had a two-folder higher odds of being diagnosed with unspecified feeding or eating disorders (40 % vs 22 % for females, *p* = 0.004). Driven exercise to control weight and shape was common and comparable in prevalence among males and females (51 % vs 47 %, *p* = 0.79) and males were less likely to present with self-induced vomiting (OR = 0.23, 95 % CI: 0.09, 0.59).

**Conclusion:**

Boys with eating disorders are an understudied group with similarities and differences in clinical presentation from girls with eating disorders. Parents and physicians are encouraged to consider changes in weight, disturbed vital signs, and driven, frequent exercise for the purposes of controlling weight or shape, as possible signs of eating disorders among male children. Diagnostic classification, assessment instruments, conceptualisation, and treatment methods need to be refined to improve application to young males.

## Background

The majority of individuals with eating disorders are female and this has generated female-centric research, with few studies examining clinical presentation among males. There is a pressing need for research on clinical presentation in males especially among boys given the rate of young males presenting for treatment (e.g., 1 in 4 children aged 5–13 years old with early onset eating disorders) [1, 2].

Males with eating disorders may have a different diagnostic distribution and course of illness than females. Bulimia nervosa (BN) was 3.8 to 4.4 times more prevalent among adolescent males than females at a tertiary clinic [3]. In tertiary settings, studies have found a four month longer duration of illness for young males than females [3] and no significant difference [4], respectively. Many factors can impact time to presentation: boys and girls presenting to tertiary treatment are typically underweight [5] which may promote earlier medical and psychiatric attention. The stigma that eating disorders are a “women’s disease” [6] may inhibit identification by primary care workers [7]. Excessive exercise is a common symptom in boys [5], and is perceived as more socially acceptable by others [8] and may be a less distressing eating disorder symptom [9]. Females may come to clinical attention sooner because they have a more extensive variety of behavioural symptoms that can be detected by others and that are subjectively distressing. Two tertiary studies found that young males had an earlier age of onset than young females [3, 10].

Widely used assessment instruments may not be well-suited to measuring cognitive symptoms in males, for instance, the Eating Disorder Examination (EDE) [11] measures shape and weight concerns associated with a desire for thinness [12], whereas some boys desire to be heavier and more muscular due to muscular-idealisation [13–16]. Girls generally favour lower weight, while boys appear to be equally split in wanting to gain or lose weight [17]. Two studies limited by small samples found that adolescent males with anorexia nervosa (AN) scored significantly lower than females on EDE weight concern and one found lower EDE shape concern [4, 18].

Medical compromise at referral to eating disorder services is common among children and adolescents because of starvation and rapid weight loss [19, 20], low body weight [19], restriction without binge eating, and less buffering adipose tissue [21]. Hypothermia has been shown to affect 33 %, bradycardia 40 %, and hypotension 20 % of children aged 5–13 years with an early-onset eating disorder [1]. It is unknown whether sex differences exist and whether young males with eating disorders in tertiary settings have a similar level or type of medical compromise and weight status to young females.

Adult-based research has found that males are more likely to excessively exercise [22–24], while females more frequently use diet pills, diuretics and laxatives and report more strict dieting, fasting, and self-induced vomiting [3, 25, 26]. This may be consistent with a tendency for males to idealise muscularity and females to idealise thinness [27, 28] which is evident in media portrayals [8]. Yet, adult-based findings may not be translatable to paediatric samples. Exercising to control weight and shape is one of the most common symptoms among children and adolescents of both sexes [5], and may be equally prevalent among girls because it is easier to undertake than fasting and purging which may be reduced because of parental oversight [29].

The aim of this study is to provide research-based insights into the clinical presentation of young males with eating disorders using the Helping to Outline Paediatric Eating Disorders (HOPE) Project registry. First, we hypothesise lower BN prevalence in males [3, 30]. Second, given mixed previous results [3, 4], we hypothesise a longer untreated illness duration for males. Third, we expect that age of onset will be lower in males [3, 10]. Fourth, we hypothesise that males will have lower shape and weight concern [4, 18]. Finally, we will explore differences in prevalence of unspecified feeding or eating disorders (UFED), medical and physical features, exercise, and self-induced vomiting.

## Methods

### Participants and procedure

The HOPE Project is an ongoing prospective clinical cohort registry for paediatric eating disorders [5] in Western Australia. The general aim of the HOPE Project is to cultivate discovery of new knowledge about eating disorders that will be of interest to the wider community, health professionals, policy-makers, and individuals affected by eating disorders. The HOPE Project registry includes children and adolescents aged 8 to 17 years who were consecutively assessed by the Child and Adolescent Mental Health Service (CAMHS) Specialised Eating Disorders Program from April 1996 (through September 2013 for this study). The CAMHS Specialised Eating Disorders Program is the only public paediatric eating disorder program in the state and offers an integrated, comprehensive continuum of care ranging from outpatient to day patient and inpatient. Referrals are accepted from general practitioners, paediatricians, psychiatrists, psychologists, school nurses, and the general public. Data originated from routine intake assessment and is detailed fully elsewhere [5]. The sample consisted of 757 consecutive referrals; *n* = 53 young males with eating disorders, *n* = 704 young females with eating disorders. Inclusion criteria for the present study were: first presentation at the service, assessment completion, and a Diagnostic and Statistical Manual (DSM-5) eating disorder diagnosis [31]. Ethical approval was granted by Princess Margaret Hospital for Children Human Research Ethics Commitee.

### Measures

Diagnosis was yielded through administration of the EDE, a gold standard semi-structured clinical interview [11]. Child and parent-informant versions of the EDE were adapted from the adult version for routine intake. Terms suitable for adults were replaced with child-appropriate terms. Clinician instructions for the laxative and diuretic misuse items were changed such that these questions were not asked if interview content up to that point suggested no further compensatory behaviours. The child-informant version used at this setting is similar but not identical to the ChEDE [32] as the service predated ChEDE publication. DSM-5 diagnosis was assigned retrospectively based on prospectively collected information from the EDE and medical review [5]. The weight criterion used for AN was expected body mass index (BMI) less than 85 %. AN, BN, binge-eating disorder (BED), and UFED could be diagnosed using the EDE. However, pica, rumination disorder, and avoidant/restrictive food intake disorder (ARFID) could not be diagnosed because the EDE did not include questions that assessed these disorders. UFED was assigned when patients presented with symptoms characteristic of a feeding or eating disorder that caused clinically significant distress or impairment in important areas of functioning and did not meet criteria for AN, BN, or BED. To establish inter-rater reliability, a trained doctoral clinical psychology student blindly diagnosed a random subset of cases in the HOPE Project cohort (*n* = 19) and agreement with diagnoses assigned by a psychologist yielded a kappa indicating substantial agreement (κ = 0.72) [33].

Duration of illness and age of onset were self-reported at the medical review by the parents and patient. Weight concern (α = 0.84, present study) and shape concern (α = 0.92, present study) were measured with the child-informant EDE [11]. BMI *z* score was calculated using the United States Centers for Disease Control and Prevention (CDC) 2000 growth reference for children and adolescents [34]. Complications associated with malnutrition were derived based on standardised vital sign data from the medical review with age-appropriate cut off points: hypothermia (body temperature [aural] <35.5 °C) [35, 36], hypotension (systolic blood pressure <90 mmHg for ≥ 10 years; <70 mmHg + [2 × age in years] for 1 to <10 years) [37], bradycardia (resting pulse <1st percentile in beats/min by age) [38], and poor peripheral perfusion (fingertip capillary refill time > 2 s) [39].

### Statistical analysis

Analyses of variance (ANOVAs) tested for differences in duration of illness, age of onset, EDE weight concern, EDE shape concern, and BMI *z* score between groups. Logistic regression models compared the odds of BN diagnosis, UFED diagnosis, self-induced vomiting, intense exercise, bradycardia, hypotension, hypothermia, and peripheral perfusion: Firth’s penalised likelihood regression was used for BN diagnosis due to small cell count. A negative binomial model was ultimately used for frequency of self-induced vomiting and a zero-inflated negative binomial model for frequency of intense exercise, as these were favoured on the basis of Akaike Information Criterion values. Unadjusted and age-adjusted parameter estimates were calculated given clinical presentation in young people can depend on age [29]; as similar results were found, unadjusted results were reported. The nominal alpha for analyses was 0.05. Although we conducted multiple tests, we sought to minimise the probability of making a Type II error in this observational study [40] thus performed no familywise error rate correction.

## Results

### Sample characteristics

Young people with eating disorders commonly presented with restricting presentations of AN or atypical AN although the most common diagnosis was UFED. Additional sample characteristics are shown in Table 1.

**Table 1:** Demographic and clinical characteristics of males and females with eating disorders in a tertiary setting

	Males	Females
	(*N* = 53)	(*N* = 704)
Age (yrs), *M* (*SD*)	13.57 (2.02)	14.74 (1.56)
DSM-5 diagnosis, *n* (%)		
UFED	21 (39.6)	153 (21.7)
AN	18 (34.0)	268 (38.1)
Atypical AN	11 (20.8)	168 (23.9)
BN	1 (1.9)	70 (9.9)
Purging disorder	1 (1.9)	28 (4.0)
BED	1 (1.9)	2 (0.3)
BN with low frequency and/or limited duration	0 (0)	10 (1.4)
BED with low frequency and/or limited duration	0 (0)	5 (0.7)
Lifetime psychiatric hospitalisations, *n* (%)	2 (4.3)	56 (9.5)
Current psychiatric medication use, *n* (%)	7 (17.9)	117 (20.4)
Age of onset (yrs), *M* (*SD*)	12.39 (2.55)	13.81 (1.66)
Illness duration (mths), median ± IQR [range]	9.00 ± 5–18 [2, 120]	8.00 ± 5–12 [1,78]
BMI *z* score, *M* (*SD*)	−1.61 (1.45)	−1.42 (1.49)
Objective binge presence, *n* (%)	9 (17.3)	137 (20.0)
Purging presence, *n* (%)	6 (11.8)	223 (32.6)
Vomiting presence	5 (9.6)	215 (30.8)
Laxatives presence	1 (1.9)	35 (5.8)
Diuretics presence	0 (0)	6 (0.9)
Intense exercise presence, *n* (%)	27 (50.9)	362 (47.3)
Objective binge episodes in the previous 28 days, median ± IQR [range]	0 ± 0–0 [0,30]	0 ± 0–0 [0, 28]
Purging episodes in the previous 28 days, median ± IQR [range]	0 ± 0–0 [0,26]	0 ± 0–6 [0, 308]
Vomiting episodes	0 ± 0–0 [0,26]	0 ± 0–4 [0,224]
Laxative episodes	0 ± 0–0 [0,10]	0 ± 0–0 [0,280]
Diuretic episodes	0 ± 0–0 [0,0]	0 ± 0–0 [0,84]
Intense exercise days in the previous 28 days, median ± IQR [range]	2.5 ± 0–28 [0,28]	4 ± 0–28 [0,28]
EDE weight concern, *M* (*SD*)	1.97 (1.50)	3.09 (1.82)
EDE shape concern, *M* (*SD*)	2.39 (1.88)	3.57 (1.81)
Any medical feature, *n* (%)	24 (45.3)	243 (34.5)
Bradycardia	16 (32.7)	156 (22.6)
Hypotension	6 (12.8)	76 (11.2)
Hypothermia	4 (8.2)	20 (3.0)
Poor peripheral perfusion	5 (10.6)	91 (13.6)

Median +/− IQR [range] were reported when variable skewness (>3) was present in 1 or more groups

*UFED* unspecified feeding and eating disorders, *AN* anorexia nervosa, *BN* bulimia nervosa, *BED* binge eating disorder, *BMI* body mass index, *EDE* Eating Disorder Examination

### Hypothesis and exploratory testing

Table 2 contains the results of the group comparisons. There were no significant differences between sexes on prevalence of BN (*p* = 0.11) or duration of illness (*p* = 0.28). Boys had a significantly younger age of onset (*p* <0.001) and lower EDE shape and weight concern (both *p*s <0.001) than girls. Boys and girls had comparable weight status and medical compromise on all features assessed. Boys had a two-fold higher odds of being diagnosed with UFED (*p* = 0.004). No significant difference was found on prevalence of exercise for shape and weight control (*p* = 0.41). Males less commonly used vomiting as a compensatory method and less frequently vomited (*p* <0.001).

**Table 2:** Differences between males and females with eating disorders on study variables

Characteristic	Mean difference	*p*	Result
Age of onset (yrs)	−1.42 (0.25)	<0.001	males < females
Duration of illness (mths)	1.12 (1.11)	0.28	
Shape concern	−1.17 (0.26)	<0.001	males < females
Weight concern	−1.11 (0.26)	<0.001	males < females
BMI *z* score	−0.22 (0.21)	0.29	
	IRR (95 % CI)	*p*	Result
Vomiting frequency	0.16 (0.06, 0.43)	<0.001	males < females
Intense exercise frequency	1.08 (0.90, 1.29)	0.41	
	OR (95 % CI)	*p*	Result
BN diagnosis	0.26 (0.05, 1.35)	0.11	
UFED diagnosis	2.36 (1.32, 4.22)	0.004	males > females
Intense exercise presence	0.93 (0.53, 1.62)	0.79	
Vomiting presence	0.23 (0.09, 0.59)	0.002	males < females
Any medical feature	0.64 (0.36, 1.12)	0.12	
Bradycardia	0.66 (0.36, 1.21)	0.18	
Hypotension	0.95 (0.39, 2.29)	0.91	
Hypothermia	0.36 (0.12, 1.09)	0.07	
Poor peripheral perfusion	1.42 (0.55, 3.67)	0.46	

*BMI* body mass index, *BN* bulimia nervosa, *CI* confidence interval, *IRR* incident rate ratio, *OR* odds ratio, *UFED* unspecified feeding or eating disorders

## Discussion

Characterising youths with eating disorders is absolutely necessary to inform identification and assessment, training for frontline primary care and allied health professionals, and treatment adaptation and planning. This study compared the clinical presentation of young males to young females with eating disorders, and extends limited, previous research [3, 4]. Differences were apparent on age of onset, cognitive symptoms, and self-induced vomiting (Table 2).

AN, atypical AN, and UFED were the most common diagnoses at this tertiary setting, consistent with findings that children and adolescents with eating disorders most commonly present with dietary restriction and food avoidance [1, 28]. Boys did not have a higher prevalence of BN, possibly because the girls in the sample had not yet traversed the age of risk for onset of BN (15–18 years) [30]. UFED, however, was significantly more common in young males than young females. This suggests that the DSM-5 diagnostic criteria may be designed more specifically for females, and may fail to capture certain symptoms that appear to be more common in males than females (e.g., muscularity-focused body image concerns, muscularity-oriented behaviours such as weight lifting). Future research is needed to explore the DSM-5 criteria to determine whether sex-related bias exists, and how to improve diagnostic classification.

Illness duration was typically less than one year and boys did not have a significantly longer duration of illness than girls. This suggests no difference in help-seeking delay between sexes for those presenting to tertiary services, possibly due to the clinical severity of those referred to tertiary services and greater parental oversight, where eating problems are detected and treated earlier [41, 42]. Energy deficits and medical compromise accompanying low body weight in paediatric patients may draw earlier medical attention than adult male patients. Young males with eating disorders had an earlier age of onset than young females, consistent with the findings in another tertiary setting [3] and that a higher proportion of pre-adolescent patients are male [19].

Young males scored lower on measures of eating disorder cognitions, specifically EDE shape and weight concern, than young females. Lower shape and weight concern could represent true lower concern (be it for weight gain or weight loss) or a measurement issue where thin-idealisation but not muscular-idealisation was the focus in assessing shape and weight concerns. The adult EDE instructions prescribe rating dissatisfaction only if respondents view their weight as too high, rather than too low, and view their shape as too large, rather than too small [11, 43]. A fairly equal proportion of boys with eating disorders seek to lose weight or gain weight, and young patients may not be able to articulate their desired body image [17]. The sex difference suggests the need for a more comprehensive assessment of shape and weight concerns among males. We recommend that clinicians ask questions relating to desire to be heavier, stronger, fitter and more muscular when interviewing young males to obtain a more accurate clinical picture of weight and shape concern, and assess the desire for control.

As expected, boys were less likely than girls to present with self-induced vomiting, even when controlling for age, consistent with adult community-based research [25]. Boys were not more likely to use exercising as a compensatory behaviour compared with girls, contrary to adult-based research [22–24]. Eating disorder populations may be atypical, for they are more likely to come from high-risk contexts involving sports such as dance, gymnastics, and athletic environments. The media has played an increasing role in promoting exercise as a socially acceptable method for weight loss and muscularity among both sexes, hence persons with these goals may adopt this practice [8].

Our study did not support a sex difference in BMI *z* scores. BMI *z* scores were below average, commensurate with other clinical samples of young people [19, 20]. Together with previous findings [1], the medical risk associated with eating disorders is emphasised by disturbances across many vital signs of participants in our sample, particularly the presence of bradycardia; and no sex differences were observed.

This study has limitations that warrant consideration. Common early onset eating disorders such as pica, rumination and ARFID could not be diagnosed. Age of onset and duration of illness were based on parent and child recall, which over a period of months may be subject to reliability problems. The small sample size of boys (7 %), particularly given comparisons to a much larger group of girls limits the generalizability of our male findings, although, such sex ratios are common in tertiary eating disorders settings and our overall sample is relatively large. More severe illness presentations are likely to be represented in a clinical rather than a community-based population because of the treatment-seeking nature of the sample, additionally, the tertiary service in this study triages on medical compromise including underweight. Hence, findings may not generalise to community-based and non-tertiary clinic populations.

## Conclusion

In conclusion, young males with eating disorders are an understudied group with similarities and differences in clinical presentation to young females. Community education efforts should alert clinicians and parents to the signs and symptoms of changes in weight, unstable vital signs, and driven exercise for the purposes of controlling weight and shape, as means to identify eating disorders in male children. Assessment instruments, diagnostic classification, and treatment applications require refinement to enhance applicability to males and the earlier age of onset finding could benefit from investigation into sex-related aetiologic and phenotypic pathways.

## Abbreviations

AN: anorexia nervosa
ARFID: avoidant/restrictive food intake disorder
BED: binge-eating disorder
BMI: body mass index
BN: bulimia nervosa
CAMHS: Child and Adolescent Mental Health Service
DSM: Diagnostic and Statistical Manual
EDE: Eating Disorder Examination
HOPE: Helping to Outline Paediatric Eating Disorders
UFED: unspecified feeding and eating disorders

## References

[1] Madden S, Morris A, Zurynski YA, Kohn M, Elliot EJ (2009). Burden of eating disorders in 5-13-year-old children in Australia. Med J Aust.

[2] Lucas AR, Beard CM, O’Fallon WM, Kurland LT (1991). 50-year trends in the incidence of anorexia nervosa in Rochester, Minn.: a population-based study. Am J Psychiatry.

[3] Eliot AO, Baker CW (2001). Eating disordered adolescent males. Adolescence.

[4] Darcy AM, Doyle AC, Lock J, Peebles R, Doyle P, Le Grange D (2012). The Eating Disorders Examination in adolescent males with anorexia nervosa: How does it compare to adolescent females?. Int J Eat Disord.

[5] Watson HJ, McCormack J, Hoiles K, Forbes D, Potts J (2013). The HOPE (Helping to Outline Paediatric Eating Disorders) Project: Development and debut of a paediatric clinical eating disorder registry. J Eat Disord.

[6] Harvey JA, Robinson JD (2003). Eating disorders in men: current considerations. J Clin Psychol Med Settings.

[7] Muise AM, Stein DG, Arbess G (2003). Eating disorders in adolescent boys: a review of the adolescent and young adult literature. J Adolesc Health.

[8] Sharp CW, Clark SA, Dunan JR, Blackwood DH, Shapiro CM (1994). Clinical presentation of anorexia nervosa in males: 24 new cases. Int J Eat Disord.

[9] Cash TF, Winstead BA, Janda LH. The great American shape-up. Psychol Today. 1986;30–37.

[10] Shafran R, Bryant-Waugh R, Lask B, Arscott K (1995). Obsessive-compulsive symptoms in children with eating disorders: a preliminary investigation. Eat Disord.

[11] Fairburn CG, Cooper Z, Fairburn CG, Wilson GT (1993). The eating disorder examination. Binge eating: nature, assessment, and treatment.

[12] Smolak L, Levine MP, Thompson JK (2001). The use of the Sociocultural Attitudes Towards Appearance Questionnaire with middle school boys and girls. Int J Eat Disord.

[13] Cohane GH, Pope HG (2001). Body image in boys: a review of the literature. Int J Eat Disord.

[14] Furnham A, Badmin N, Sneade I (2002). Body image dissatisfaction: Gender differences in eating attitudes, self-esteem, and reasons for exercise. J Psychol.

[15] Parkinson K, Tovee M, Cohen-Tovée E (1998). Body shape perceptions of preadolescent and young adolescent children. Eur Eat Disord Rev.

[16] Adams K, Sargent RG, Thompson SH, Richter D, Corwin SJ, Rogan TJ (2000). A study of body weight concerns and weight control practices of 4th and 7th grade adolescents. Ethn Health.

[17] Furnham A, Calnan A (1998). Eating disturbance, self-esteem, reasons for exercising and body weight dissatisfaction in adolescent males. Eur Eat Disord Rev.

[18] Strober M, Freeman R, Lampert C, Diamond J, Teplinsky C, DeAntonio M (2006). Are there gender differences in core symptoms, temperament, and short-term prospective outcome in anorexia nervosa?. Int J Eat Disord.

[19] Peebles R, Wilson JL, Lock JD (2006). How do children with eating disorders differ from adolescents with eating disorders at initial evaluation?. J Adolesc Health.

[20] Le Grange D, Crosby RD, Rathouz PJ, Leventhal BL (2007). A randomized controlled comparison of family-based treatment and supportive psychotherapy for adolescent bulimia nervosa. Arch Gen Psychiatry.

[21] Birmingham CL, Treasure J. Medical management of eating disorders*.* Cambridge University Press; 2010.

[22] Crisp A, Burns T (1983). The clinical presentation of anorexia nervosa in males. Int J Eat Disord.

[23] Margo JL (1987). Anorexia nervosa in males. A comparison with female patients. Br J Psychiatry.

[24] Lewinsohn PM, Seeley JR, Moerk KC, Striegel-Moore RH (2002). Gender differences in eating disorder symptoms in young adults. Int J Eat Disord.

[25] Anderson CB, Bulik CM (2004). Gender differences in compensatory behaviors, weight and shape salience, and drive for thinness. Eat Behav.

[26] Braun DL, Sunday SR, Huang A, Halmi KA (1999). More males seek treatment for eating disorders. Int J Eat Disord.

[27] Ricciardelli LA, McCabe MP (2004). A biopsychosocial model of disordered eating and the pursuit of muscularity in adolescent boys. Psychol Bull.

[28] McCreary DR, Sasse DK (2000). An exploration of the drive for muscularity in adolescent boys and girls. J Am Coll Health.

[29] Walker T, Watson HJ, Leach DL, McCormack J, Tobias K, Hamilton MJ, Forbes DA (2014). Comparative study of children and adolescents referred for eating disorder treatment at a specialist tertiary setting. Int J Eat Disord.

[30] Carlat DJ, Camargo CA (1991). Review of bulimia nervosa in males. Am J Psychiatry.

[31] American Psychiatric Association (2013). Diagnostic and statistical manual of mental disorders (DSM-5).

[32] Bryant-Waugh RJ, Cooper P, Taylor CL, Lask BD (1996). The use of the Eating Disorder Examination with children: a pilot study. Int J Eat Disord.

[33] Landis JR, Koch GG (1977). The measurement of observer agreement for categorical data. Biometrics.

[34] Kuczmarski RJ, Ogden CL, Grummer-Strawn LM, Flegal KM, Guo SS, Mei Z (2000). CDC Growth Charts: United States. Adv Data.

[35] Brooke OG (1972). Influence of malnutrition on the body temperature of children. Br Med J.

[36] World Health Organization (1999). Management of severe malnutrition: a manual for physicians and other senior health workers.

[37] Kleinman ME, Chameides L, Schexnayder SM, Samson RA, Hazinski MF, Atkins DL, Berg MD, de Caen AR, Fink EL, Freid EB (2010). Pediatric advanced life support: 2010 American Heart Association Guidelines for Cardiopulmonary Resuscitation and Emergency Cardiovascular Care. Pediatrics.

[38] Moseley BD, Nickels K, Britton J, Wirrell E (2010). How common is ictal hypoxemia and bradycardia in children with partial complex and generalized convulsive seizures?. Epilepsia.

[39] Bumke K, Maconochie I (2001). Paediatric capillary refill times. Trauma.

[40] Rothman KJ (1990). No adjustments are needed for multiple comparisons. Epidemiology.

[41] Arnow B, Sanders MJ, Steiner H (1999). Premenarcheal versus postmenarcheal anorexia nervosa: a comparative study. Clin Child Psychol Psychiatry.

[42] Fisher M, Schneider M, Burns J, Symons H, Mandel FS (2001). Differences between adolescents and young adults at presentation to an eating disorders program. J Adolesc Health.

[43] Fairburn CG (2008). Cognitive behavior therapy and eating disorders.

